# Identification of *M.tuberculosis*-Specific Th1 Cells Expressing CD69 Generated *in vivo* in Pleural Fluid Cells from Patients with Tuberculous Pleurisy

**DOI:** 10.1371/journal.pone.0023700

**Published:** 2011-08-22

**Authors:** Li Li, Dan Qiao, Xiaoying Fu, Suihua Lao, Xianlan Zhang, Changyou Wu

**Affiliations:** 1 Institute of Immunology, Zhongshan School of Medicine, Key Laboratory of Tropical Disease Control Research of Ministry of Education, Sun Yat-sen University, Guangzhou, People's Republic of China; 2 Chest Hospital of Guangzhou, Guangzhou, People's Republic of China; Charité-University Medicine Berlin, Germany

## Abstract

Th1 cell-mediated immune responses at the site of active infection are important to restrict the growth of *M.tuberculosis* (MTB) and for the spontaneous resolution of patients with tuberculous pleurisy (TBP). In the present study, we found that without any stimulation, CD4^+^ T cells in pleural fluid cells (PFCs) from patients with TBP expressed significantly higher levels of CD69 than PBMCs from patients with tuberculosis (TB) or healthy donors. CD4^+^CD69^+^ T cells expressed T-bet and IL-12Rβ2. After stimulation with MTB-specific antigens, CD4^+^CD69^+^ T cells expressed significantly higher levels of IFN-γ, IL-2 and TNF-α than CD4^+^CD69^−^ T cells, demonstrating that CD4^+^CD69^+^ T cells were MTB-specific Th1 cells. In addition, CD4^+^CD69^+^ T cells were mostly polyfunctional Th1 cells that simultaneously produced IFN-γ, IL-2, TNF-α and displayed an effector or effector memory phenotype (CD45RA^−^CCR7^−^CD62L^−^CD27^−^). Moreover, the percentages of CD4^+^CD69^+^ T cells were significantly and positively correlated with polyfunctional T cells. Interestingly, sorted CD4^+^CD69^+^ but not CD4^+^CD69^−^ fractions by flow cytometry produced IFN-γ, IL-2 and TNF-α that were significantly regulated by CD4^+^CD25^+^ Treg cells. Taken together, based on the expression of CD69, we found a direct quantitative and qualitative method to detect and evaluate the *in vivo* generated MTB-specific polyfunctional CD4^+^ T cells in PFCs from patients with TBP. This method can be used for the potential diagnosis and enrichment or isolation of MTB-specific Th1 cells in the investigations.

## Introduction

Tuberculous pleurisy (TBP) is characterized by an intense chronic accumulation of inflammatory cells at the disease site, including the activation of Th1 cells and their preferential recruitment to the affected areas [1], [2]. However, TBP remains difficult to identify despite numerous diagnostic tools [3]. Currently, the definite diagnosis of tuberculosis (TB) pleural effusions depends on the demonstration of acid-fast bacilli in the sputum, pleural fluid, or pleural biopsy specimens [4]. Moreover, high levels of IFN-γ are detected in tuberculosis pleural fluid [5], [6]. Therefore, measurement of IFN-γ in the pleural fluid has also gained wide acceptance in the diagnosis of TB pleural effusions [7]. However, these assays did not provide information regarding antigen-responsive cells. An abundance of immunocompetent cells in the pleural effusion provides investigators with a good model to study the correlates of a protective immune response at the site of infection. The enrichment of Th1 cells in pleural fluid has been previously documented [8]. However, detailed phenotypic and functional characterizations for these cells are still unknown.

The BCG vaccine is an attenuated strain of *Mycobacterium bovis* and includes loss of the esx 1 locus, which encodes two family members, culture filtrate protein of 10 kDa (CFP-10) and early secretory antigenic target 6 (ESAT-6). These antigens have an important diagnostic role in distinguishing BCG vaccination from MTB infection [9]–[13]. Although ESAT-6 and CFP-10 contain numerous potential T cell epitopes, the immune response during infection is often focused toward a few immunodominant epitopes. In the present study, we selected the six most dominant peptides identified in ESAT-6 and CFP-10, as reported to be frequently recognized by subjects in Indian, European, Cambodian, and Middle Eastern cohorts with both latent and active TB [14]–[19].

It has already been demonstrated that T-lymphocytes in the lungs, both in normal individuals and in those with granulomatous disease, are almost entirely CD45RO^+^ memory T cells and express both early- and late-activation markers, such as CD69 and CD29 respectively [20]. Previous data have demonstrated that almost all of the CCR5^+^ and CCR3^+^CD4^+^ T cells recruited to the lungs of patients with active TB express the memory T-cell phenotype and that the majority may have been recently activated [21]. CD69 is a membrane molecule transiently expressed on activated lymphocytes, and its selective expression in inflammatory infiltrates probably suggests that it plays a role in the pathogenesis of inflammatory diseases [22]. Previous studies have also demonstrated the usefulness of the determination of CD69 on CD4^+^ T cells after in vitro stimulation with tuberculin as a rapid indicator of immune sensitization against MTB [23], [24]. However, these studies did not directly assess the function of CD69^+^ cells.

The present study was initially designed to analyze the discrepancy of phenotypes among pleural fluid cells (PFCs) from patients with TBP, PBMCs from TB patients and normal donors. We found a significant increase in CD69 expression on CD4^+^ T cells in PFCs. Importantly, CD4^+^CD69^+^ cells were mostly Th1 cells specific for ESAT-6 and CFP-10 peptides. The phenotypic and functional analysis of CD69-expressing cells strongly suggested that CD69 could be a useful marker for the identification or enrichment of antigen specific Th1 cells at local sites following MTB infection. CD69 may be useful for diagnostic procedures, including specific immune monitoring during TB infection or after vaccination, as well as for therapeutic applications such as targeted adoptive Th1 cell therapies.

## Results

### Significantly higher expression of CD69 on CD4^+^ T cells in PFCs from patients with TBP than in PBMCs from TB patients and healthy donors without any stimulation

**Figure 1:**
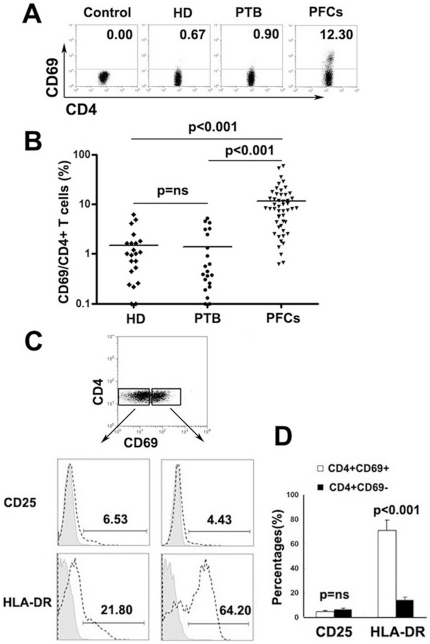
Significantly higher expression of CD69 on CD4^+^ T cells in PFCs from patients with TBP without any stimulation. (A) Representative expression of CD69 on CD4^+^ T cells was determined by FACS in PBMCs from healthy donors (HD), patients with pulmonary tuberculosis (PTB) and pleural fluid cells from patients with TBP (PFCs). (B) Summary data of CD69 expression on HD (n = 22), PTB (n = 21) and PFCs (n = 50). Each symbol represents a value from a single donor. Horizontal lines denote mean expression. (C) Expression of CD25 and HLA-DR on CD4^+^CD69^+^ and CD4^+^CD69^−^ T cells. The expression of each marker within the CD4^+^CD69^+^ and CD4^+^CD69^−^ T cell subsets is shown by the dotted line. (D) Average value of CD25 and HLA-DR expression on CD4^+^CD69^+^ and CD4^+^CD69^−^ T cells from six independent experiments is demonstrated. ns, not significant.

Our initial experiments assessed the surface expression of CD69 on CD4^+^ T cells from PFCs of patients with TBP and PBMCs from TB patients (PTB) and healthy donors (HD). As expected, without any stimulation, a significantly higher level of CD69 expression was observed on CD4^+^ T cells from PFCs of TBP patients than on PBMCs from either PTB or HD (Fig. 1A and B, P<0.001). Moreover, higher levels of CD69 expression (11.60%±11.93%, mean ± SD) on CD4^+^ T cells were observed in 78% of the PFCs from individuals with TBP than on the PBMCs of PTB or HD (1.48%±1.60% for healthy donors and 1.38%±1.68% for TB patients, mean ± SD). No significant difference in CD69 expression was observed on PBMCs from PTB and from HD (Fig 1B, P = ns). In addition, we also demonstrated that CD4^+^CD69^+^ T cells expressed significantly higher levels of HLA-DR than CD4^+^CD69^−^ T cells, a molecule which is usually induced on CD4^+^ T cells after activation (Fig 1C and D, P<0.001). Unexpectedly, no significant difference was observed in the expression of CD25, another activation marker, which is usually temporally expressed on activated T cells or constitutively expressed on regulatory T cells (Fig 1C and D, P = ns).

### Significantly higher expression of T-bet and IL-12Rβ2 by CD4^+^CD69^+^ T cells than by CD4^+^CD69^−^ T cells

**Figure 2:**
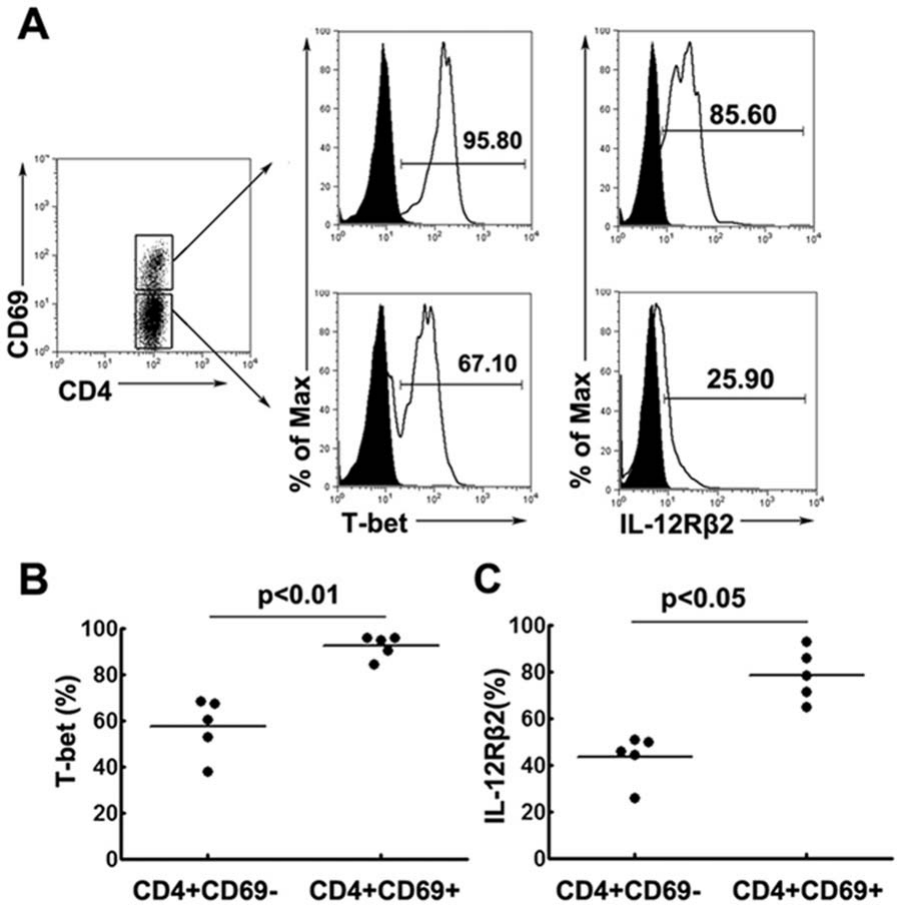
Expression of T-bet and IL-12Rβ2 by CD4^+^CD69^+^ and CD4^+^CD69^−^ T cells as assessed by direct ex vivo staining. (A) Expression of T-bet and IL-12Rβ2 was evaluated on CD4^+^CD69^+^ and CD4^+^CD69^−^ T cells by flow cytometry. (B, C) Summary data of T-bet (B) and IL-12Rβ2 (C) expression on CD4^+^CD69^+^ and CD4^+^CD69^−^ T cells derived from five independent experiments are represented.

In order to confirm whether CD4^+^CD69^+^ T cells in PFCs were Th1 cells, we further evaluated the expression of T-bet, an important transcription factor specific for Th1 cells. The results showed that more than 90% of CD4^+^CD69^+^ T cells and about 57.24% of CD4^+^CD69^−^ T cells expressed T-bet (Fig 2A). The expression of T-bet on CD4^+^CD69^+^ T cells was significantly higher than that on CD4^+^CD69^−^ T cells (Fig 2B, P<0.01). We next examined the expression of IL-12Rβ2, another marker highly specific for differentiated Th1 cells. Similar to the results obtained for T-bet expression, the majority of CD4^+^CD69^+^ T cells constitutively expressed IL-12Rβ2 (Fig. 2A), and the expression was significantly higher than that observed on CD4^+^CD69^−^ T cells (Fig 2C, P<0.05 ), further confirming that the CD4^+^CD69^+^ T cell subset was highly enriched for Th1 cells.

### ESAT-6 and CFP-10 peptides-specific Th1 cells were preferentially enriched within CD4^+^CD69^+^ T cells

**Figure 3:**
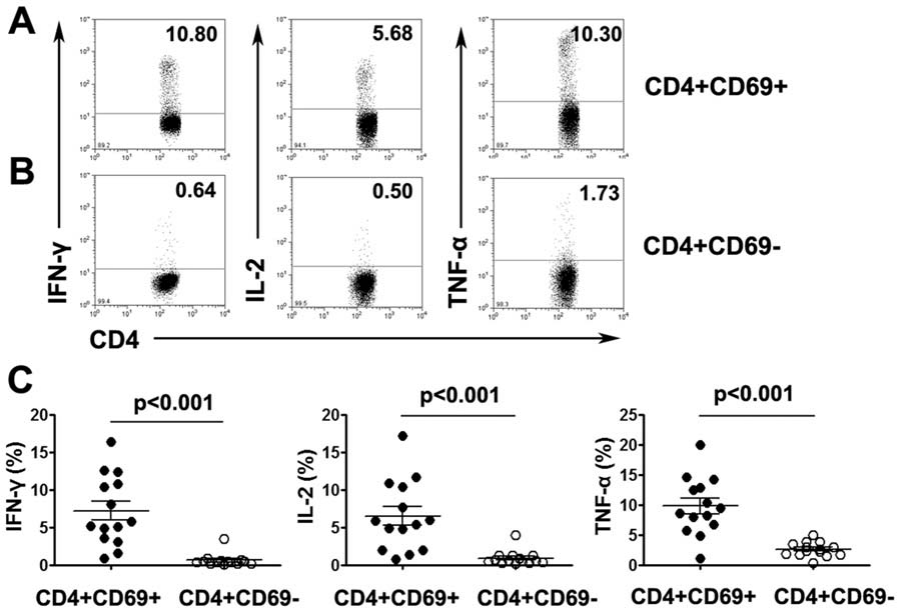
Th1 cytokine-producing cells were enriched within CD4^+^CD69^+^ T cells. (A, B) PFCs were stimulated with ESAT-6/CFP-10 peptides (P1-P6), anti-CD28mAbs, anti-CD49d mAbs and BFA for eight hours. Cells were gated on CD4^+^CD69^+^ and CD4^+^CD69^−^ T cell populations. Representative expression of IFN-γ, IL-2 and TNF-α on CD4^+^CD69^+^ (A) and CD4^+^CD69^−^ T cells (B) is shown. The numbers in each quadrant represent the percentages of positive cells in gated T cells. (C) Summary data of the frequency of cytokine-producing CD4^+^CD69^+^ and CD4^+^CD69^−^ T cells (n = 14). Horizontal lines represent means ± SD.

Because CD4^+^CD69^+^ T cells were predominantly Th1 cells, we aimed to examine whether direct *ex vivo* evaluation of surface expression of CD69 without *in vitro* upregulation permits direct assessment of MTB-specific Th1 cells. Our initial experiments indicated that stimulation of PFCs with ESAT-6 and CFP-10 peptides resulted in increased surface CD69 expression that is difficult to elucidate (Data not shown). Short-term stimulation of these cells includes incubation with secretion inhibitory brefeldin A (BFA), which can block the transport of newly synthesized CD69 molecules to the cell surface and thus allows for the direct assessment of the relationship between *in vivo* CD69 expression and cytokine production. After culturing PFCs with ESAT-6/CFP-10 peptides and BFA, we analyzed the production of Th1 cytokines by intracellular cytokine staining and polychromatic flow cytometry. As shown in Fig 3A and B, CD4^+^CD69^+^ T cells produced greater amounts of IFN-γ, IL-2 and TNF-α than did CD4^+^CD69^−^ T cells. Statistical results demonstrated that the percentages of IFN-γ, IL-2 or TNF-α-producing CD4^+^CD69^+^ T cells were significantly higher than those for CD4^+^CD69^−^ T cells (P<0.001, Fig 3C). In order to further confirm that the responses of T cells were specific for MTB, we also stimulated PFCs from tuberculous pleurisy patients with other irrelevant antigens such as SARS-CoV S peptides (STFFSTFKCYGVSATKL) and (NFSQILPDPLKPTK RSFI) as well as hepatitis B virus surface antigen (HBsAg). We found that these irrelevant antigens could not induce the production of IFN-γ, IL-2 and TNF-α by neither CD4^+^CD69^+^ nor CD4^+^CD69^−^ T cells. However, following stimulation with MTB-specific peptides of ESAT-6/CFP-10, CD4^+^CD69^+^ T cells from the same patients expressed high levels of IFN-γ, IL-2 and TNF-α (data not shown).

### Significant positive correlation between the percentages of CD69-expressing CD4^+^ T cells and polyfunctional T cells

**Figure 4:**
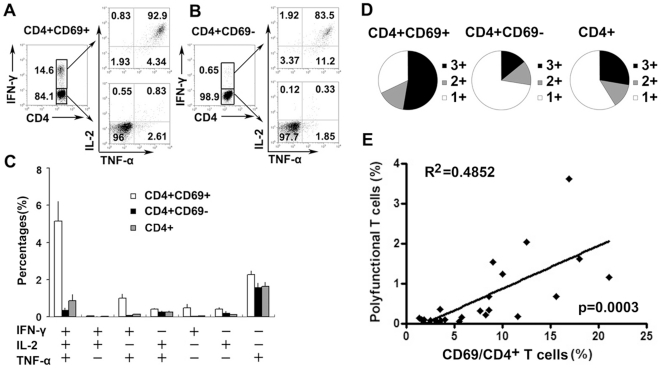
Significantly positive correlation between the percentages of CD4^+^CD69^+^ T cells and polyfunctional T cells. (A) Cells were gated on CD4^+^CD69^+^IFN-γ^+^ and CD4^+^CD69^+^IFN-γ^−^ cells, respectively. Representative expression of TNF-α and IL-2 within gated cells is shown. (B) Cells were gated on CD4^+^CD69^−^IFN-γ^+^ and CD4^+^CD69^−^IFN-γ^−^ cells, respectively. The representative expression of TNF-α and IL-2 within gated cells is shown. The numbers in each quadrant represent the percentages of positive cells in gated T cells. (C) The average frequencies of CD4^+^CD69^+^, CD4^+^CD69^−^ and CD4^+^ T cells expressing each of the seven combinations of IFN-γ, IL-2 and TNF-α from twenty-two independent experiments are demonstrated. The mean and SEM are shown. (D) Responses are grouped according to the number of functions. The pie charts summarize the fractions of single (1+ blank), double (2+ gray), and triple (3+ black) producers of IFN-γ, IL-2 and TNF-α within the CD4^+^CD69^+^, CD4^+^CD69^−^ and CD4^+^ T cell subsets from twenty-two independent experiments. (E) Significant positive correlation between CD4^+^CD69^+^ T cells and polyfunctional T cells (p = 0.0003, n = 22).

It has been suggested that T cells simultaneously producing IFN-γ, IL-2 and TNF-α are associated with protective immunity and concomitant beneficial outcomes, at least in chronic viral infections such as HIV. We therefore compared the expression of IFN-γ, IL-2 and TNF-α in CD4^+^CD69^+^, CD4^+^CD69^−^ and total CD4^+^ T cells after short-term *in vitro* stimulation with ESAT-6/CFP-10 peptides. We showed that CD4^+^CD69^+^ and CD4^+^CD69^−^ T cells concurrently and individually produced IFN-γ, IL-2 and TNF-α (Fig 4A, B). This assay also identified cells that expressed CD69 but lacked expression of any cytokine measured (as evidenced by a subset of CD4^+^CD69^+^IFN-γ^−^IL-2^−^TNF-α^−^ cells). These cells probably contained other effector functions, for example, the secretion of MIP-1β or other cytokines. The total antigen-specific responses were defined as the percentages of T cells expressing any combination of IFN-γ, IL-2 or TNF-α, and thus, seven distinct functional populations could be delineated (IFN-γ/IL-2/TNF-α triple expressers; IFN-γ/IL-2, IFN-γ/TNF-α or TNF-α/IL-2 double expressers; or IFN-γ, IL-2 or TNF-α single expressers). Interestingly, polyfunctional T cells (IFN-γ/IL-2/TNF-α triple expressers) dominated the CD4^+^CD69^+^ T cell response. However, frequencies of T cells producing only TNF-α were the highest when analyzing the distribution of the seven subsets within either the CD4^+^CD69^−^ population or all CD4^+^ T cells (Fig 4C, D). The percentages of double expressers were similar within CD4^+^CD69^+^, CD4^+^CD69^−^ and total CD4^+^ T cells. Our results demonstrated that total MTB-specific CD4^+^ T cells were not dominated by polyfunctional T cells. However, polyfunctional T cells were primarily enriched within CD4^+^CD69^+^ T cells. We further analyzed the correlation between the percentages of CD69-expressing CD4^+^ T cells and polyfunctional T cells. Interestingly, significant positive correlation between CD4^+^CD69^+^ T cells and polyfunctional T cells was observed (Fig 4E, p = 0.0003).

### CD4^+^CD69^+^ T cells were mostly effector memory T cells

**Figure 5:**
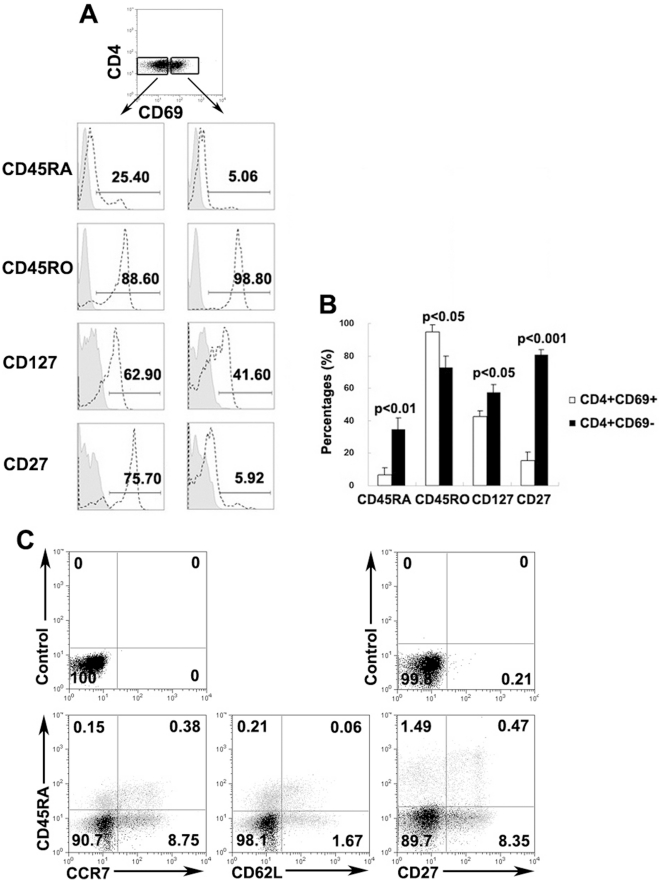
Effector memory CD4^+^CD69^+^ T cells produced IFN-γ in response to MTB-specific peptides. (A) PFCs were stained with CD4, CD69, CD45RA, CD45RO, CD127 and CD27. The expression of each marker within CD4^+^CD69^+^ and CD4^+^CD69^−^ T cells was evaluated by FACS. Isotype controls are shown in gray and the expression of each marker is shown by dotted lines. (B) Mean value of expression of each marker on CD4^+^CD69^+^ and CD4^+^CD69^−^ T cells. Differences between groups were assessed by the Wilcoxon matched pairs test (Two-tailed). (C) PFCs stimulated with P1-P6 were gated on CD4^+^CD69^+^IFN-γ^+^ and CD4^+^CD69^+^IFN-γ^−^ cells and analyzed for the expression of memory markers CD45RA, CCR7, CD62L and CD27, as indicated. Upper panel represents the control. Lower panel represents the expression of each marker. CD4^+^CD69^+^IFN-γ^+^ T cells are shown in black, whereas CD4^+^CD69^+^IFN-γ^−^ T cells are depicted in gray. The expression of each marker within CD4^+^CD69^+^IFN-γ^+^ T cells was overlaid with CD4^+^CD69^+^IFN-γ^−^ T cells. The numbers in the quadrants indicate the percentage of cells among CD4^+^CD69^+^IFN-γ^+^ T cells. One representative result from ten independent experiments with similar results is shown.

We next investigated the phenotype of CD4^+^CD69^+^ T cells relating to naïve/memory markers. As shown in Fig 5A, CD4^+^CD69^+^ T cells were predominantly CD45RO^+^CD45RA^−^, thereby exhibiting a memory cell phenotype. In contrast, CD4^+^CD69^−^ T cells contained significantly higher percentages of CD45RA^+^ and lower percentages of CD45RO^+^ cells than did CD4^+^CD69^+^ T cells. Moreover, we also examined the expression of CD127 and CD27 and found that CD4^+^CD69^+^ T cells had significantly lower expression of both markers (Fig 5B). To further validate whether MTB-specific CD4^+^CD69^+^ T cells were memory cells, we tested the phenotype of IFN-γ-secreting CD4^+^CD69^+^ T cells by assessing typical memory T cell markers. As expected, the majority of CD4^+^CD69^+^IFN-γ^+^ T cells displayed a CD45RA^−^CCR7^−^CD62L^−^CD27^−^ effector memory cell phenotype (Fig 5C), suggesting that these cells were capable of producing multiple cytokines quickly at local sites.

### CD69 expression identified viable MTB-specific Th1 cells

**Figure 6:**
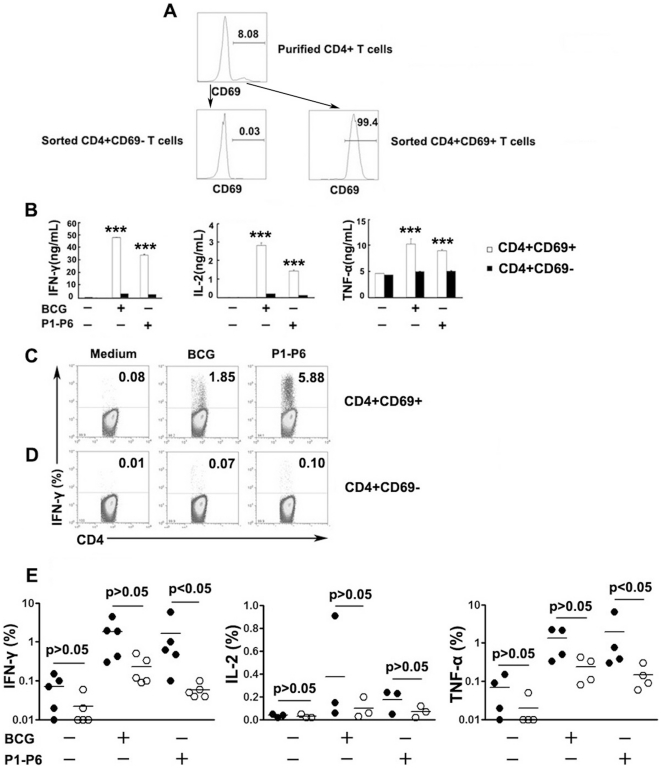
Isolation of viable CD4^+^CD69^+^ and CD4^+^CD69^−^ T cells. (A) CD4^+^CD69^+^ and CD4^+^CD69^−^ cells were sorted from magnetic bead-purified CD4^+^ T cells. (B) Sorted CD4^+^CD69^+^ and CD4^+^CD69^−^ cells were cocultured with purified CD14^+^ cells in the presence or absence of BCG or P1-P6. Supernatants were collected and cytokine production was assessed by ELISA. Significant differences compared with CD4^+^CD69^−^ fractions are indicated (***, P<0.001). (C, D) After stimulation for 8 hours, CD4^+^ T cells were gated and the expression of Th1 cytokines by CD4^+^CD69^+^ and CD4^+^CD69^−^ cells was analyzed. Numbers in the quadrants indicate percentages of cells among the CD4^+^CD69^+^ or CD4^+^CD69^−^ populations. (E) The frequencies of IFN-γ, IL-2 or TNF-α-producing CD4^+^CD69^+^ (closed circles) or CD4^+^CD69^−^ T cells (open circles, n = 3–5) are demonstrated. Horizontal lines represent mean value.

We further investigated whether purified CD4^+^CD69^+^ T cells, stained by fluorescently labeled monoclonal antibody and sorted by flow cytometry, would be compatible with assays that required live cells. We first purified CD4^+^ T cells and CD14^+^ cells by positive selection with microbeads. Thereafter, CD4^+^CD69^+^ and CD4^+^CD69^−^ T cell fractions were obtained by flow cytometry-based cell sorting (Fig 6A). We then cocultured CD4^+^CD69^+^ or CD4^+^CD69^−^ T cells with CD14^+^ cells (as antigen-presenting cells) in the presence of ESAT-6/CFP-10 peptides (P1–P6) and measured the production of Th1 cytokines. Notably, considerable levels of IFN-γ and IL-2 were produced by CD4^+^CD69^+^ T cells, whereas little was observed from the CD4^+^CD69^−^ fractions. However, TNF-α production was readily detected in unstimulated cells, and significantly higher TNF-α was measured following stimulation of the CD4^+^CD69^+^ fractions (Fig 6B). Moreover, significantly higher levels of IFN-γ, IL-2 and TNF-α were produced by CD4^+^CD69^+^ cells than by CD4^+^CD69^−^ T cells (P<0.001, Fig 6B). We also assessed percentages of Th1 cytokine-producing cells by flow cytometry. As expected, CD4^+^CD69^+^ T cells were highly enriched for IFN-γ, IL-2 and TNF-α production compared with CD4^+^CD69^−^ cells (Fig 6C–E). Thus, CD69 expression provides a means to identify the vast majority of antigen-specific CD4^+^ T cells, regardless of the type of cytokine measured. Importantly, our assay conditions based on the CD69 coculture assay retained the ability of cells to secrete multiple cytokines.

### CD4^+^CD25^+^ T cells inhibit production of Th1 cytokines by CD4^+^CD69^+^ T cells

**Figure 7:**
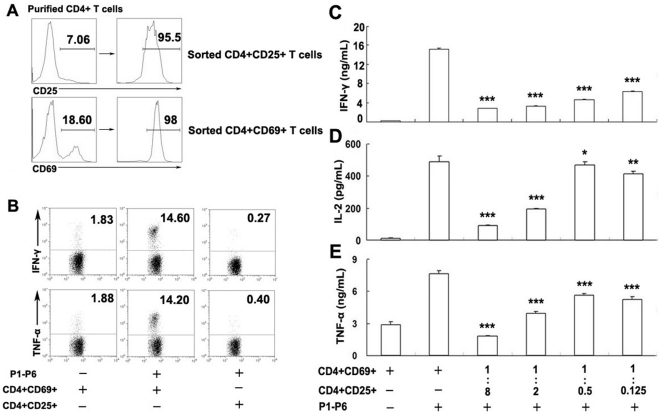
CD4^+^CD25^+^ cells inhibit the production of Th1 cytokines by sorted CD4^+^CD69^+^ T cells. (A) CD4^+^CD25^+^ and CD4^+^CD69^+^ cells were sorted from purified CD4^+^ T cells. (B) Production of IFN-γ and TNF-α by sorted CD4^+^CD69^+^ and CD4^+^CD25^+^ cells in the presence of purified CD14^+^ T cells following stimulation with P1–P6. (C–E) Sorted CD4^+^CD25^+^ and CD4^+^CD69^+^ cells were cultured at different ratios and cocultured with purified CD14^+^ cells in the presence or absence of P1–P6. The supernatants were collected, and cytokine production was assessed by ELISA. One representative result from three independent experiments with similar results is shown. Significant differences compared with incubation with P1–P6 in the absence of Tregs are indicated (*, not significant;**, P<0.05;***, P<0.001).

In order to examine whether CD4^+^CD25^+^ Treg cells could directly inhibit cytokine production by CD4^+^CD69^+^ T cells, we sorted CD4^+^CD69^+^ and CD4^+^CD25^+^ T cells by flow cytometry (Fig 7A). In order to further confirm that CD4^+^CD25^+^ T cells were regulatory T cells, we found that the majority of CD4^+^CD25^+^ T cells (>95%) expressed Foxp3(Data not shown), the specific marker for this subset. We further confirmed this observation by incubation of sorted CD4^+^CD69^+^ or CD4^+^CD25^+^ T cells with CD14^+^ T cells, respectively. The result demonstrated that neither IFN-γ nor TNF-α was produced by the CD4^+^CD25^+^ T cells, whereas CD4^+^CD69^+^ T cells produced large amounts of IFN-γ and TNF-α upon coculture (Fig 7B), confirming that CD4^+^CD25^+^ T cells were hyporesponsive and represented regulatory T cells. We then cocultured CD4^+^CD69^+^ with CD4^+^CD25^+^ T cells at different ratios in the presence of CD14^+^ cells. CD4^+^CD69^+^ cells stimulated with ESAT-6/CFP-10 peptides (P1–P6) produced considerably greater amounts of Th1 cytokines compared with unstimulated cells. The addition of CD4^+^CD25^+^ T cells into cultures resulted in the inhibition of IFN-γ, IL-2 and TNF-α production in a dose-dependent manner (Fig 7C–E). Overall, our assay conditions showed that CD4^+^CD25^+^ cells could inhibit Th1 cytokine production by CD4^+^CD69^+^ T cells.

## Discussion

Without treatment, tuberculous pleurisy (TBP) usually resolves spontaneously [25] and thus is thought to be a useful model to study the correlates of protective immune responses at the site of infection[26], [27]. Meanwhile, the diagnosis of TBP is difficult and continues to pose clinical challenges. Currently, the two major advances in the diagnosis of TBP have been pleural biopsies[4] and adenosine deaminase (ADA) assessment in pleural fluid [28], [29]. The free (unstimulated) pleural fluid IFN-γ assay is another method established for the diagnosis of TBP[30]. However, both ADA and IFN-γ are non-specific markers of inflammation[30].

The detection of T cell responses specific for defined antigens is essential for the evaluation of pathogenic or protective immunity induced by infectious pathogens or vaccines. CD4^+^ T cell responses are commonly determined by the expression of various cytokines, such as IFN-γ, IL-2 and TNF-α for Th1 cells, after short-term stimulation with specific antigens. The development of IFN-γ release assays (IGRAs) has emerged as an attractive alternative to the tuberculin skin test for detection of latent TB infections and might also contribute to assisting in the diagnosis of TBP [31]. IGRAs are based on the principle that T cells of individuals sensitized with TB antigens produce IFN-γ when they re-encounter highly MTB-specific antigens such as ESAT-6 and CFP-10. However, this assay precludes further analysis because it is lethal to cells. Moreover, the IGRAs are not recommended on either the blood or the pleural fluid for the diagnosis of TBP [31].

Consistent with other studies, we found that the expression of CD69 was significantly increased on CD4^+^ T cells in PFCs from patients with TBP [32]. We further demonstrated that the majority of CD4^+^CD69^+^ cells were Th1 cells by analyzing expression of the Th1 markers T-bet and IL-12Rβ2. In order to further confirm that CD4^+^CD69^+^ cells were mostly MTB-specific T cells, we performed intracellular cytokine staining. We noted that stimulation of PFCs resulted in increased surface CD69 expression. We overcame this limitation by incubation with BFA during short-term stimulation. Using this method, we were able to directly assess the relationship between in vivo CD69 expression and cytokine production. Using polychromatic flow cytometry, we demonstrated for the first time that the majority of MTB specific Th1 cytokine-producing cells were enriched within the CD4^+^CD69^+^ subset, whereas CD4^+^CD69^−^ cells scarcely produced cytokines. In addition, PFCs from tuberculous pleurisy patients were stimulated with SARS-CoV S peptides (STFFSTFKCYGVSATKL) and (NFSQILPDPLKPTKRSFI) as well as hepatitis B virus surface antigen (HBsAg). These irrelevant antigens could not induce the production of IFN-γ, IL-2 and TNF-α by neither CD4^+^CD69^+^ nor CD4^+^CD69^−^ T cells. However, following stimulation with MTB-specific peptides of ESAT-6/CFP-10, CD4^+^CD69^+^ T cells from the same patients with tuberculous pleurisy expressed high levels of IFN-γ, IL-2 and TNF-α, indicating that the response was MTB-specific. Most importantly, the detection of CD69 is useful for the direct identification of MTB-specific Th1 cells *in vivo* without further stimulation.

T cells that produce multiple factors simultaneously are termed polyfunctional T cells and have been shown to provide vaccine-induced immunity and protection against disease progression in HIV-1 infection[33], [34]. In the present study, analysis of total CD4^+^ Th1 subpopulations indicated that the majority of Th1 cells produced only one cytokine. However, polyfunctional CD4^+^ T cells simultaneously producing IFN-γ, IL-2 and TNF-α dominated the CD4^+^CD69^+^ T cell population, but the percentage of TNF-α single expressers was higher in both the CD4^+^CD69^−^ and the total CD4^+^ T cell subsets compared with CD4^+^CD69^−^ cells. Our results indicated that detection or isolation of CD69^+^ T cells is a good method for the identification or enrichment of polyfunctional T cells. Interestingly, significant positive correlation was observed between the percentages of CD69-expressing CD4^+^ T cells and polyfunctional T cells. Our results demonstrated for the first time that the expression of CD69 may be a useful marker for the definition of MTB-specific polyfunctional T cells in vivo. A number of studies in humans suggested that polyfunctional T cells may indeed be involved in mediating protection in TB[34]–[39]. However, it has also been indicated in the recent studies that polyfunctional CD4^+^ T cells may be a useful biomarker of active TB and that polyfunctional responses are not associated with protection against TB[40]. In the present study, we hypothesized that the highly increased frequencies of CD69-expressing CD4^+^ T cells (polyfunctional T cells) in pleural fluid may be associated with protection against TB and thus lead to the self-resolution tendency of TBP.

A previous study has indicated that CD4^+^CD69^+^CD25^−^ cells represented a new subset of regulatory T cells in the tumor model [41]. These CD4^+^CD69^+^CD25^−^ cells are hyporesponsive and can suppress proliferation of CD4^+^ T cells in a cell-to-cell contact manner. In our study, however, CD4^+^CD69^+^ T cells represented effector or effector memory cells and quickly produced large amounts of cytokines following short-term stimulation. Moreover, we also assessed other markers (Foxp3 and CTLA-4) essential for the suppressive function of regulatory T cells and found that these molecules were not differentially expressed by CD4^+^CD69^+^ and CD4^+^CD69^−^ T cells, further confirming that CD4^+^CD69^+^ T cells were not regulatory T cells (data not shown). These differing results were probably associated with discrepancies in local circumstances, including cytokines, chemokines and antigen presenting cells that differ between tumor and inflammation contexts. Moreover, it remains to be determined if CD69 is induced by the pleural fluid environment or if CD4^+^CD69^+^ T cells are selectively recruited from the circulation.

Previous studies have indicated that CD4^+^CD25^+^ T cells can inhibit antigen-specific T cell responses in TB patients[42]–[47]. However, none of these studies has isolated MTB-specific CD4^+^ T cells directly. In the present study, we purified viable CD4^+^CD69^+^ and CD4^+^CD25^+^ Treg cells directly and found that CD4^+^CD25^+^ Treg cells could inhibit cytokine production by CD4^+^CD69^+^ T cells. In addition, our results clearly demonstrated that CD4^+^CD25^+^ T cells were hyporesponsive and scarcely produced cytokines following short-term stimulation, further confirming that these cells were regulatory cells. In our previous studies, we have demonstrated that the suppressive effect of Treg cells on IFN-γ production by CD4^+^CD25^+^ T cells[43] and γδ T cells[48] was mainly mediated via the release of IL-10 but not TGF-β.

In summary, we demonstrated that CD69 as a useful marker for MTB-specific Th1 cells in PFCs from patients with TBP enabled a direct *ex vivo* estimation of the quantity, as well as the quality, of MTB-specific Th1 responses. The majority of CD4^+^CD69^+^ T cells but not total CD4^+^ T cells, were dominated by polyfunctional T cells. Importantly, significantly positive correlation was observed between CD69-expressing CD4^+^ T cells and polyfunctional T cells, suggesting that CD69 is a good marker for the identification or enrichment of polyfunctional T cells in PFCs. Moreover, isolation of live MTB-specific Th1 cells according to surface CD69 expression without any stimulation offers an opportunity for further investigation.

## Methods

### Ethics Statement

Written informed consent was obtained from all patients and healthy donors. Ethics approval for the present study was obtained from the ethics committee of the Zhongshan School of Medicine, Sun Yat-sen University (Guangzhou, China) and the Chest Hospital of Guangzhou (Guangzhou, China).

### Study participants

A total of fifty patients with tuberculous pleurisy (15 females and 35 males, range 18–71 years of age) were recruited from the Chest Hospital of Guangzhou, China. Diagnosis of pleural effusion from TB etiology was based on one of the following criteria: (i) demonstration of MTB on pleural fluid smear (by the Ziehl-Neelsen method); (ii) pleural fluid or pleural biopsy specimens growing *M. tuberculosis* on Lowenstein-Jensen medium; or (iii) histological evidence of caseating granuloma on biopsy specimens of pleural tissue with positive staining for *M. tuberculosis*. Twenty-one patients (10 females and 11 males, range 20 to 50 years of age) with active pulmonary tuberculosis (culture-confirmed pulmonary TB) were also included in this study. Patients who had been previously diagnosed with HIV, HBV, or HCV or with a history of autoimmune diseases were excluded from the study. None of the patients was receiving MTB-related treatments at the time of the study. Twenty-two healthy volunteers between the ages of 20 and 54 were recruited from Sun Yat-sen University.

### Peptides, reagents and mAbs

For the detection of MTB-specific immune responses, we selected six highly immunogenic and largely HLA-DR-restricted peptides. Four peptides were derived from ESAT-6, and two were derived from the CFP-10 protein of MTB. Synthetic peptides of 20 amino acids (aa) in length were obtained from Shenzhen Hanyu manufacture, Shenzhen, China. The sequences of the peptides are as follows: P1, ESAT-6_1–20_ (MTEQQWNFAGIEAAASAIQG); P2, ESAT-6_31–50_ (EGKQSLTKLAAAWGGSGSEA); P3, ESAT-6_61–80_ (TATELNNALQNLARTISEAG); P4, ESAT-6_71–90_ (NLARTISEAGQAMASTEGNV); P5, CFP-10_51–70_ (AQAAVVRFQ EAANKQKQELD) and P6, CFP-10_71–90_ (EISTNIRQAGVQYSRADEEQ). Freeze-dried BCG (Chengdu Institute of Biological Products, Chengdu, China) was reconstituted in a 0.9% sodium chloride solution to a concentration of 1 mg/mL prior to use. Purified anti-CD28 (clone CD28.2) and anti-CD49d (clone 9F10) mAbs were purchased from BD Biosciences (San Jose, CA). The following mAbs were used for phenotypic and intracellular cytokine analysis and were purchased from BD Biosciences (San Jose, CA, USA): CD62L-PE (Dreg56), CCR7-PE (3D12), TNF-α-PE (Mab11), CD69-PE (FN50), T-bet-PE (O4-46), Streptavidin-PE, CD45RA-FITC (L48), CD25-FITC (M-A251), CD4-PerCP (L200), CD8-Pecy7 (RPA-T8), CD69-Pecy7 (FN50), APC-IL-2 (MQ1-17H12), purified rat anti-human CD212 (2B6/12β2), and isotype-matched control antibodies. CD27-APC (O323) was obtained from Biolegend (San Diego, CA). IFN-γ-FITC (45.15) was purchased from Beckman Coulter (Fullerton, CA). Biotin-SP-conjugated affinipure F(ab')_2_ fragment donkey anti-rat IgG (H+L) was purchased from JacksonImmunoResearch laboratories, Inc. (West Grove, PA).

### Preparation of PFCs and PBMCs

PFCs were isolated by lysing erythrocytes using ammonium chloride solution and resuspended to a final concentration of 2×10^6^ cells/mL in complete RPMI 1640 medium (Invitrogen, Grand Island, NY) supplemented with 10% heat-inactivated fetal calf serum (FCS; HyClone, Logan, UT), 100 U/mL penicillin, 100 µg/mL streptomycin, 2 mM L-glutamine, and 50 µM 2-mercaptoethanol. Peripheral blood mononuclear cells (PBMCs) were isolated by Ficoll-Hypaque gradient centrifugation of heparinized venous blood obtained from healthy individuals or TB patients.

### Flow cytometry

For the detection of intracellular cytokines, cells were incubated at a concentration of 2×10^6^ cells/mL with 1 µg/mL peptides plus 1 µg/mL anti-CD28 and 1 µg/mL anti-CD49d for 8 h in the presence of brefeldin A (BFA, 10 µg/mL; Sigma-Aldrich, St Louis, MO). For the detection of T-bet, surface markers were first stained and cells were then fixed in 2% formaldehyde, permeabilized in 90% methanol and labeled with anti T-bet mAb. The expression of IL-12Rβ2 was detected by indirect immunofluorescence analysis. Briefly, cells were incubated with mAb for IL-12Rβ2, followed by biotin-SP-conjugated anti-rat IgG F(ab')_2_ fragments, and were finally incubated with streptavidin-PE. All incubations were performed at 4°C and cells were washed twice between incubations. Flow cytometry was performed using BD FACSCalibur (BD Biosciences) or FACSAria II (BD Biosciences) and the data were analyzed using FlowJo software (TreeStar, San Carlos, CA).

### Isolation of cell subsets

CD4^+^ and CD14^+^ cells were isolated from PFCs by positive selection with anti-CD4 or anti-CD14 microbeads (Miltenyi Biotec, Bergisch Gladbach, Germany). For isolation of CD4^+^CD69^+^, CD4^+^CD69^−^ and CD4^+^CD25^+^ cells, purified CD4^+^ T cells were stained with PE-conjugated CD69 and FITC-conjugated anti-CD25 and then sorted on a FACSAria II high-speed cell sorter. The purity of cells, as assessed by flow cytometry, was 95–97% for each cell subset.

### Cell culture conditions

Purified CD4^+^CD69^+^, CD4^+^CD69^−^ or CD4^+^ T cells were co-cultured with purified CD14^+^ cells (at ratio of 1∶2 ) in the presence or absence of BCG, ESAT-6 and CFP-10 peptides. After 48 hours, cell-free supernatants were collected and assessed for IFN-γ, IL-2 and TNF-α production by ELISA kits (BD Bioscience Pharmingen). For the detection of intracellular cytokines, cells were cultured for 8 hours and then subjected to flow cytometry analysis. To examine the role of CD4^+^CD25^+^ cells in the regulation of cytokine production by sorted CD4^+^CD69^+^ T cells, purified CD4^+^CD69^+^ (Teff), CD14^+^ cells and CD4^+^CD25^+^ cells (Treg) were co-cultured in various ratios (Teff:Treg) in the presence of ESAT-6 and CFP-10 peptides. Cell-free supernatants were collected and assessed for IFN-γ, IL-2 and TNF-α by ELISA.

### Statistical analysis

The Wilcoxon matched pairs test (Two-tailed) was performed to determine statistical differences between the groups using GraphPad Prism software version 5. Inhibition of cytokine production by CD4^+^CD25^+^ T cells was analyzed using the paired Student's t test. A value of *p*<0.05 was considered statistically significant.
